# BOPPPS-driven learning plan guidance in preventive medicine laboratory education: Implementation strategy and multidimensional outcomes

**DOI:** 10.1186/s12909-026-10038-8

**Published:** 2026-07-28

**Authors:** Wei Mi, Ziyi Lv, Yao Song, Ming Su, Qingshuo Meng, Lifeng Xiao, Binpeng Zhang, Jia Kong, Sha Liu

**Affiliations:** 1 School of Public Health, Binzhou Medical University, Yantai, 264003 China; 2 Second School of Clinical Medicine, Binzhou Medical University, Yantai, 264003 China

**Keywords:** BOPPPS, Learning plan guidance, Preventive medicine, Laboratory education

## Abstract

**Background:**

Conventional preventive medicine laboratory training often overemphasizes procedural replication, leaving analytical reasoning and emergency response skills underdeveloped. To address this, a BOPPPS strategy (bridge-in, objective, pre-assessment, participatory learning, post-assessment, summary) was implemented via learning plan guidance. This cluster randomized controlled trial study evaluated its dual impact on learner capability and instructional quality.

**Methods:**

Participants included undergraduates enrolled in 2022 from Binzhou Medical University’s Clinical Medicine and Medical Imaging programs, divided into an intervention group receiving BOPPPS-driven learning plan guidance and a control group receiving traditional instruction. Multidimensional assessments combined laboratory performance with validated instruments: the Schommer Epistemological Questionnaire (SEQ), Opinions about Learning Survey (OLS), Motivated Learning Strategies Questionnaire (MLSQ), and Teacher Self-Efficacy Scale (TSES). Structural equation modeling analyzed relationships among epistemological beliefs, learning perspectives, and strategies.

**Results:**

The intervention group achieved significantly higher total lab scores than controls. SEQ results showed superior scores in knowledge stability, structure, and authority. OLS indicated stronger preference for structure and certainty. MLSQ revealed higher deep-learning strategy scores. Structural modeling identified positive links between epistemological beliefs, learning perspectives, and surface strategies; knowledge stability and preference for structure positively correlated with deep strategies, while omniscient authority and preference for certainty showed negative correlations. TSES scores reflected high educator efficacy in student management, teaching strategies and classroom management.

**Conclusion:**

The BOPPPS-driven learning plan guidance significantly enhanced experimental performance and cultivated self-regulated learning, innovation, and critical thinking. Simultaneously, they improved instructional efficiency and effectiveness, offering a replicable model for competency-based training in preventive medicine education.

**Supplementary Information:**

The online version contains supplementary material available at https://doi.org/10.1186/s12909-026-10038-8.

## Introduction

Preventive medicine occupies a central role in modern healthcare and public health systems, with laboratory education serving as a critical pillar for translating theoretical knowledge into practical competencies and cultivating the analytical mindset required of public health professionals. Aligned with the global shift toward competency-based medical education, preventive medicine laboratory curricula are increasingly expected to foster not only technical proficiency but also self-regulated learning, critical reasoning, and innovative problem-solving abilities that support lifelong professional development. Nevertheless, preventive medicine laboratory education in many settings remains dominated by traditional didactic, teacher-centered models. Instructors typically rely on lecture-based demonstrations that prioritize procedural replication and outcome verification, while giving insufficient attention to the underlying logic of experimental design and methodological reasoning [1].

Consequently, students often focus narrowly on obtaining expected experimental results and completing laboratory reports, with limited engagement with core principles, experimental troubleshooting, or biosafety risk management—deficiencies that undermine the development of core competencies for public health practice [2, 3]. Previous pedagogical reforms, including problem-based learning [4], flipped classroom approaches [5] and self-directed experimental design [6] have shown preliminary benefits in enhancing student engagement and scientific reasoning. However, most existing initiatives adopt isolated instructional strategies rather than a systematic, full-cycle strategy tailored to the unique demands of preventive medicine laboratory training, leaving a gap between current pedagogical practices and the competency goals of modern medical education.

The BOPPPS instructional strategy, which originates from Canadian teacher competency development programs, comprises six sequential phases: bridge-in (B), objective (O), pre-assessment (P), participatory learning (P), post-assessment (P), and summary (S), forming the BOPPPS acronym. This model establishes an iterative feedback cycle within didactic processes through learner-centered participatory engagement and dynamic interaction-feedback mechanisms, demonstrating globally validated pedagogical efficacy [7]. It is grounded in two core principles: goal-aligned instructional design and a learner-centric epistemology. Centered on participatory learning as its operational core, the strategy facilitates problem-based discourse supported by real-time feedback, thereby fostering self-regulated learning agency that enhances academic engagement and multidimensional competency. Educators guide curricular development and instructional iteration across a six-phase continuum, directing case-based collaborative inquiry to cultivate competencies in problem identification and resolution, while constructing integrated knowledge frameworks. This approach optimizes didactic effectiveness through cognitive scaffolding [8]. The model has gained increasing prominence in medical education, with documented pedagogical improvements across multiple disciplines, including thoracic surgery [9], physiology [10], nursing [11], clinical hematological testing techniques [12], and ophthalmology [13]. As an adaptive instructional architecture, the BOPPPS strategy synergizes multi-modal pedagogies to align with medical students’ cognitive-behavioral profiles, capitalizing on its inherent flexibility to optimize knowledge integration and clinical reasoning development [14]. Wen et al. demonstrated the synergistic integration of the BOPPPS with case-based learning (CBL) in nursing electrocardiogram education [15], which stimulated learner engagement (cognitive-affective domain) and enhanced instructional quality optimization through innovative curriculum scaffolding, establishing cross-disciplinary pedagogical hybridization principles. Concurrently, Javaheri et al. conceptualized a novel GW (guided walkthrough)-BOPPPS integration in medical pedagogy [16], capitalizing on the model’s inherent adaptability—particularly its dynamic participatory learning architecture—to amplify student motivation through context-responsive cognitive activation. Despite these strengths, standalone BOPPPS implementation primarily focuses on in-class instructional design, with limited systematic support for pre-class autonomous preparation and post-class knowledge extension—two critical components of laboratory education that require sustained learning outside of class time.

As an outcome of China’s pedagogical modernization initiatives, the learning plan guidance model has established a scalable implementation framework for student-centered educational paradigms. This teacher-facilitated, learner-driven approach utilizes structured learning protocols as cognitive mediators to develop autonomous learning competencies through scaffolded instructional strategies. It systematically promotes critical analysis and adaptive problem-solving abilities, which are essential for the development of multidimensional competencies. The learning plan guidance pedagogy comprises four core phases: guided pre-class self-study, in-class collaborative problem-solving, integrated knowledge construction, and formative feedback and reflection. This approach is designed to systematically build autonomous learning competencies and higher-order thinking skills by shifting the responsibility for knowledge construction to learners [17].

Recently, the learning plan guidance model has gained considerable traction in medical education [17], with successful implementation reported in areas such as preventive medicine [18], neurobiology [19], and nursing practice training [20]. Although the term “learning plan guidance” is not explicitly documented in international literature, comparable pedagogical approaches that emphasize self-regulated learning (SRL) competencies are prevalent, aligning with the strategic emphasis in medical education on fostering lifelong learning proficiency [21, 22]. Alt et al. advocate for pedagogical adaptations that support engaged, self-directed knowledge acquisition [23], while educators enhance their instructional design capacities through theory-practice integrated curricular development—a viewpoint reinforced by Khalil and Elkhider, who underscore the importance of pedagogical engineering for optimizing learning outcomes [24]. However, the model often lacks standardized, structured in-class interaction and assessment protocols, which can lead to inconsistent implementation and variable achievement of learning objectives—particularly in laboratory settings where operational standardization and real-time feedback are critical. The BOPPPS strategy and learning plan guidance model share a foundational alignment with learner-centered, competency-based educational philosophy, creating strong theoretical potential for synergistic integration. Functionally, the two models are highly complementary: the structured six-phase BOPPPS architecture provides a standardized, feedback-driven roadmap for in-class laboratory sessions, addressing the implementation inconsistency of standalone learning plan guidance. In turn, the learning plan guidance model extends the BOPPPS cycle with systematic pre-class preparation and post-class reflection scaffolds, addressing the limited out-of-class support of standalone BOPPPS. Together, they form a complete “pre-class – in-class – post-class” learning cycle that matches the full workflow of preventive medicine laboratory education, and aligns with the core goals of modern medical education to build both technical and cognitive competencies. Educators empirically assess instructional effectiveness using validated psychometric instruments—including the Schommer Epistemological Questionnaire (SEQ), Opinions about Learning Survey (OLS), Motivated Learning Strategies Questionnaire (MLSQ), and Teacher Self-Efficacy Scale (TSES)—to monitor cognitive-behavioral trajectories and refine model implementation through evidence-based iteration [25].

To date, no published studies have investigated the integrated application of the BOPPPS strategy and learning plan guidance model in preventive medicine laboratory education, and there is a lack of empirical evidence for full-cycle, competency-focused pedagogical reform in this specific context. This study therefore developed and implemented a BOPPPS-driven learning plan guidance model for preventive medicine laboratory courses, and evaluated its multidimensional impacts on undergraduate students’ cognitive mastery, self-regulated learning competencies, and critical thinking skills. The findings aim to provide an evidence-based, actionable implementation framework for preventive medicine laboratory education reform, and to support the cultivation of high-quality public health talent equipped to meet evolving healthcare demands.

## Methods

### Participants

This cluster randomized controlled trial involved undergraduate students enrolled in 2022 from Binzhou Medical University’s Clinical Medicine (New Medical Science Class) and Medical Imaging (Classes 1–2). Intact classes were adopted as independent clusters, and cluster randomization was implemented. Each class was divided into two subgroups by random selection to assign subgroups. One subgroup from each class was randomly assigned to the control group, and the remaining subgroup to the intervention group. Finally, three subgroups were allocated to the control group and three to the intervention group. Since cluster randomization may introduce bias from intraclass correlation, intraclass correlation coefficients (ICC) were adjusted in all statistical analyses to eliminate the potential confounding influence of within-group correlation, and all baseline indicators were balanced between groups (*P* > 0.05). Students in both the intervention and control groups completed three questionnaires, namely the SEQ, OLS and MLSQ. Teachers of the intervention group filled out the TSES. In addition, the two groups received identical teaching arrangements including class hours and experimental modules. Written informed consent was obtained from all participants prior to the commencement of the study. To evaluate potential confounding factors, baseline characteristics—including sex, age, pre-intervention theoretical test scores, pre-intervention practical operation scores, pre-study learning interest score and pre-intervention learning motivation scores—were compared both between the intervention and control groups and between the two academic majors (Clinical Medicine vs. Medical Imaging) to ensure comparability prior to the intervention.

Sample size and power analysis: A priori sample size estimation and statistical power analysis were performed for this cluster randomized controlled trial, where intact classes served as independent clusters. A two-sided significance level of *α* = 0.05 and target statistical power of 0.80 were pre-specified. The intra-cluster correlation coefficient (ICC) was set at 0.07 based on published studies of classroom-based educational interventions. Key pre-defined parameters covered the primary outcome measure, expected between-group difference, standard deviation of the primary outcome, an average cluster size of 32 students per class, and an anticipated 15% loss-to-follow-up rate. The design effect (DE) attributable to within-cluster correlation was calculated using the formula (*DE* = 1 + (*m* − 1) × *ICC*), which produced a design effect of 3.17 after substitution of all parameters.

### Instructional model design

The combination of BOPPPS and learning plan guidance is theoretically grounded in constructivism. BOPPPS standardizes structured in-class interactive sessions, while learning plan guidance acts as a pre-class scaffold to arrange preview tasks. The two approaches compensate for each other’s defects: learning plans activate autonomous pre-class learning, and BOPPPS deepens knowledge internalization via classroom participation, forming an integrated pre-class and in-class teaching system consistent with modern medical education concepts.

This pedagogical intervention was applied to six experimental modules within preventive medicine laboratory education: three focused on experimental operations—disinfection of domestic drinking water, sanitary quality testing of fresh milk, and determination of whole blood cholinesterase activity—and three on analytical evaluations—design and evaluation of nutritional diets, internship in epidemiological methodology, and case discussions on occupational and food poisoning incidents. Both the control and intervention cohorts completed these six modules within a standardized curricular strategy consisting of 18 contact hours (40 min per class hour). Three qualified instructors were formally trained prior to implementation and assigned to three separate classes, with each instructor delivering instruction to both the intervention subgroup and control subgroup of their designated class. A standardized instructor training protocol was implemented to ensure consistent understanding and execution of teaching procedures across all trainers. Strict intervention fidelity monitoring was conducted throughout the whole teaching period, including regular classroom observations and standardized teaching checklists, to minimize implementation bias and guarantee uniform delivery of content, teaching pace, and total instructional hours between the two groups. Both cohorts followed an identical six-day teaching schedule with perfectly matched total instructional time.

The control group received conventional laboratory instruction, whereas the intervention group implemented BOPPPS-driven learning plan guidance. The teaching procedure implemented in the control group is detailed as follows.

Pre-class phase: Teachers provide competency-aligned learning objectives, key content points, and annotated PowerPoint presentations to scaffold preparatory self-directed learning.

In-class implementation: Teachers deliver systematic theoretical instruction using blackboard writing or PowerPoint presentations. For instance, prior to the “case discussions of food poisoning” experiment, pre-analytical didactic sessions cover diagnostic criteria, emergency protocols, and therapeutic interventions. Additionally, instructors emphasize common errors and necessary precautions to enhance students’ experimental success rates.

Post-instructional phase: Students engaged in laboratory experiments categorized into two modalities: three technical skill modules requiring collaborative task execution within assigned groups, and three analytical reasoning modules in which instructors presented case studies using PowerPoint, accompanied by guided questioning to facilitate structured group discussion and analysis. Each session concluded with a summative debriefing that addressed core conceptual complexities and operational challenges.

Post-laboratory assessment: Students are required to submit individual experimental reports, which are assessed by instructors based on predefined evaluation criteria (Table S1). Teachers grade the assignments and provide corresponding feedback.

**Fig. 1:**
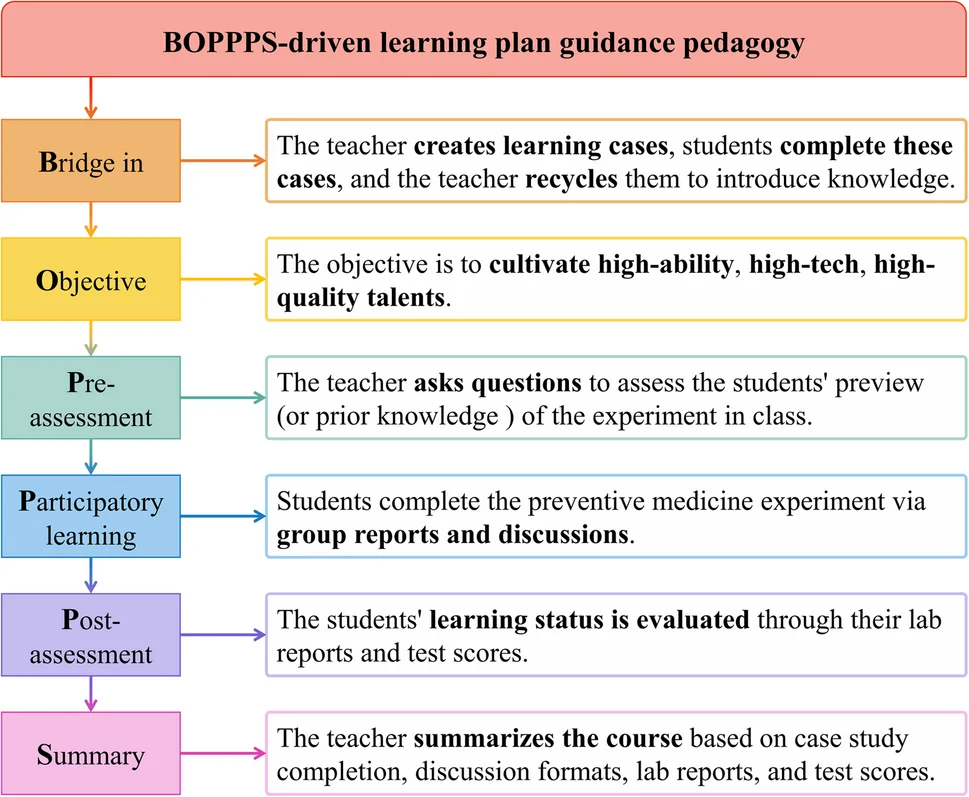
Operational workflow diagram of the BOPPPS-driven learning plan guidance pedagogy in preventive medicine laboratory education

The intervention group received standardized teaching guided by the BOPPPS-driven learning plan pedagogy (Fig. 1). The intervention spanned six formal teaching days, with each 120-minute laboratory session adopting a fixed, stage-based time allocation for the six BOPPPS modules, allowing a minor time deviation of ± 2 min: Bridge-in (15 min), Objective (10 min), Pre-assessment (15 min), Participatory Learning (60 min), Post-assessment (15 min), and Summary (5 min). To ensure standardized and replicable implementation, a complete food poisoning case-based learning system was uniformly developed, covering pre-class preview frameworks, targeted pre-class questions, structured group discussion tasks, in-class formative assessments, and post-class extended assignments, together with unified archived discussion record sheets. All instructors completed an 8-hour pre-service standardized training, including theoretical interpretation of the BOPPPS model and simulated teaching drills; only instructors who passed the unified assessment were qualified to deliver formal teaching. Throughout the intervention, an independent supervisor monitored teaching fidelity using a self-designed BOPPPS Implementation Consistency Checklist for each session. All learning plans, classroom audio recordings, and monitoring checklists were systematically archived, and weekly teaching reflection meetings were conducted to revise and standardize non-compliant teaching behaviors. Multiple rigorous control measures were implemented to minimize implementation variability across the three instructors, including identical teaching materials, timer-based duration regulation, reciprocal classroom observation, unified scoring rubrics, and consistent in-class Q&A criteria, forming a fully traceable teaching archive system. Quantitative monitoring results verified high implementation fidelity, with an overall compliance rate of 97.2% and individual instructor compliance rates ranging from 96.5% to 98.1%, confirming excellent cross-teacher consistency and ensuring high reproducibility of the educational intervention.

### Effectiveness evaluation

At the conclusion of the course, instructors graded the experimental reports based on predefined evaluation criteria. Questionnaires assessing epistemological beliefs (SEQ), learning conceptions (OLS), and learning strategies (MLSQ) were administered to students in both the intervention and control groups via the *Duifene* WeChat platform. Additionally, instructors in the intervention group completed the Teacher TSES) to self-assess instructional efficacy. All questionnaires were adapted from previously published research with appropriate modifications [25].

#### Evaluation of experimental scores

Assessment in preventive medicine laboratory education encompasses six experimental modules, evaluated across three weighted dimensions: completion of experiments (60%), emphasizing adherence to experimental procedures and accuracy of results; analysis of experimental results (20%), focusing on logical coherence and relevance of data interpretation; and standardization of reports (20%), assessing organizational clarity and adherence to documentation standards.

#### SEQ

The SEQ assesses three belief dimensions: the stability of knowledge, the structure of knowledge, and omniscient authority. Each belief dimension encompasses distinct items to assess individuals’ perceptions of knowledge and their epistemic stances comprehensively. Assessment in preventive medicine laboratory education encompasses six experimental modules, evaluated across three weighted dimensions: completion of experiments (60%), emphasizing adherence to experimental procedures and accuracy of results; analysis of experimental results (20%), focusing on logical coherence and relevance of data interpretation; and standardization of reports (20%), assessing organizational clarity and adherence to documentation standards. The SEQ has been widely employed in medical education research, with established validity and reliability in health science student populations [26].

#### OLS

The OLS comprises two subscales: preference for structure and preference for certainty. The questionnaire employs a 5-point Likert scale, where 1 represents “completely unable to do” and 5 represents “completely able to do”. This instrument was developed to assess individuals’ conceptions of teaching and learning. In his 2011 study, Aypay adapted the questionnaire and investigated its association with epistemological beliefs—a line of inquiry that bears direct relevance to our research [27].

#### MLSQ

The MLSQ comprises two subscales: deep learning strategies (evaluating critical thinking) and surface learning strategies (emphasizing rote memorization). This self-reported assessment instrument employs a 5-point Likert scale ranging from 1 (‘nonproficient’) to 5 (‘highly proficient’). Cronbach’s *α* coefficients for the subscales were 0.72 (deep learning) and 0.66 (surface learning). The MSLQ has been widely used in medical education research to assess motivational orientations and learning strategies. Its validity has been confirmed across diverse medical student populations [28].

#### TSES

The TSES serves as a validated assessment tool for evaluating instructional feedback efficacy. In this study, teaching outcomes within the intervention group were specifically assessed, without intergroup comparisons. Responses were quantified using a 5-point proficiency scale: 1 = completely unable to do, 2 = mostly unable to do, 3 = partially able to do, 4 = mostly able to do, and 5 = completely able to do. Psychometric analysis revealed high internal consistency across subdomains (Cronbach’s *α* = 0.711–0.862), exceeding thresholds for educational intervention research. Cronbach’s *α* coefficients range from 0.711 to 0.862. Originally developed and validated by Tschannen-Moran and Woolfolk Hoy, the TSES has demonstrated robust psychometric properties across cultures. Recent studies have further confirmed its reliability in medical education contexts [29].

### Statistical methods

The measurement data are expressed as the means ±standard deviations (‾*x* ± *s*). Intergroup comparisons were conducted via independent *t* tests. Structural equation modeling (SEM) and Monte Carlo mediation analysis (MCMAM) were utilized to examine the relationships among epistemological beliefs, learning conceptions, and learning strategies within the intervention group. Data analysis was conducted using Prism 6 (GraphPad), SPSS 27.0, and AMOS 23.0, with the significance level set at *α* = 0.05.

## Results

### Baseline characteristics and group comparability

To evaluate potential biases stemming from the randomization scheme and the enrollment of students from two distinct majors, baseline characteristics were compared from two dimensions. As presented in Table 1, the intervention and control groups showed no statistically significant differences regarding age (*t* = 0.593, *P* = 0.554), sex distribution (*χ²* = 0.072, *P* = 0.788), pre-intervention theoretical test scores (*t* = 0.366, *P* = 0.714), pre-intervention practical operation scores (*t* = 0.583, *P* = 0.561), pre-study learning interest scores (*t* = 0.572, *P* = 0.569) and pre-intervention learning motivation scores (*t* = 0.716, *P* = 0.475). Additionally, pairwise comparisons in Table 2 revealed that all baseline indicators were balanced between Clinical Medicine and Medical Imaging majors (all *P* > 0.05). These findings confirm that academic major did not act as a notable confounding variable, and satisfactory baseline comparability was achieved at both group and major levels.

**Table 1:** Comparison of baseline characteristics between the intervention and control groups

Baseline indicators	Control group (*n* = 61)	Intervention group (*n* = 60)	t/χ²-value	*P*-value	Cohen’s d	95% CI for mean difference
Age (years)	19.26 ± 1.08	19.14 ± 1.13	0.593	0.554	0.109	[-0.278, 0.518]
Sex (male/female)	27/34	25/35	0.072	0.788		
Pre-intervention theoretical test scores	78.35 ± 6.42	77.91 ± 6.15	0.366	0.714	0.070	[-1.823, 2.703]
Pre-intervention practical operation scores	76.28 ± 7.06	75.53 ± 6.84	0.583	0.561	0.108	[-1.752, 3.252]
Pre-study learning interest score	32.64 ± 4.18	32.17 ± 4.33	0.572	0.569	0.110	[-1.062, 2.002]
Pre-intervention learning motivation score	45.72 ± 5.36	44.98 ± 5.12	0.716	0.475	0.140	[-1.147, 2.627]

**Table 2:** Comparison of baseline characteristics between students from Clinical Medicine and Medical Imaging majors

Baseline indicators	Clinical medicine (*n* = 40)	Medical imaging (*n* = 81)	t/χ²-value	*P*-value	Cohen’s d	95% CI for mean difference
Age (years)	19.33 ± 1.12	19.21 ± 1.09	0.574	0.567	0.120	[-0.296,0.536]
Sex (male/female)	18/22	34/47	0.041	0.840		
Pre-intervention theoretical test scores	78.51 ± 6.37	77.84 ± 6.22	0.551	0.583	0.107	[-1.693,3.033]
Pre-intervention practical operation scores	76.45 ± 7.12	75.70 ± 6.93	0.530	0.597	0.107	[-1.764,3.264]
Pre-study learning interest score	32.81 ± 4.25	32.24 ± 4.21	0.693	0.490	0.135	[-1.067,2.207]
Pre-intervention learning motivation score	45.94 ± 5.41	45.07 ± 5.27	0.842	0.401	0.164	[-1.081,2.721]

### Comparative analysis of experimental scores between the control and intervention groups

**Fig. 2:**
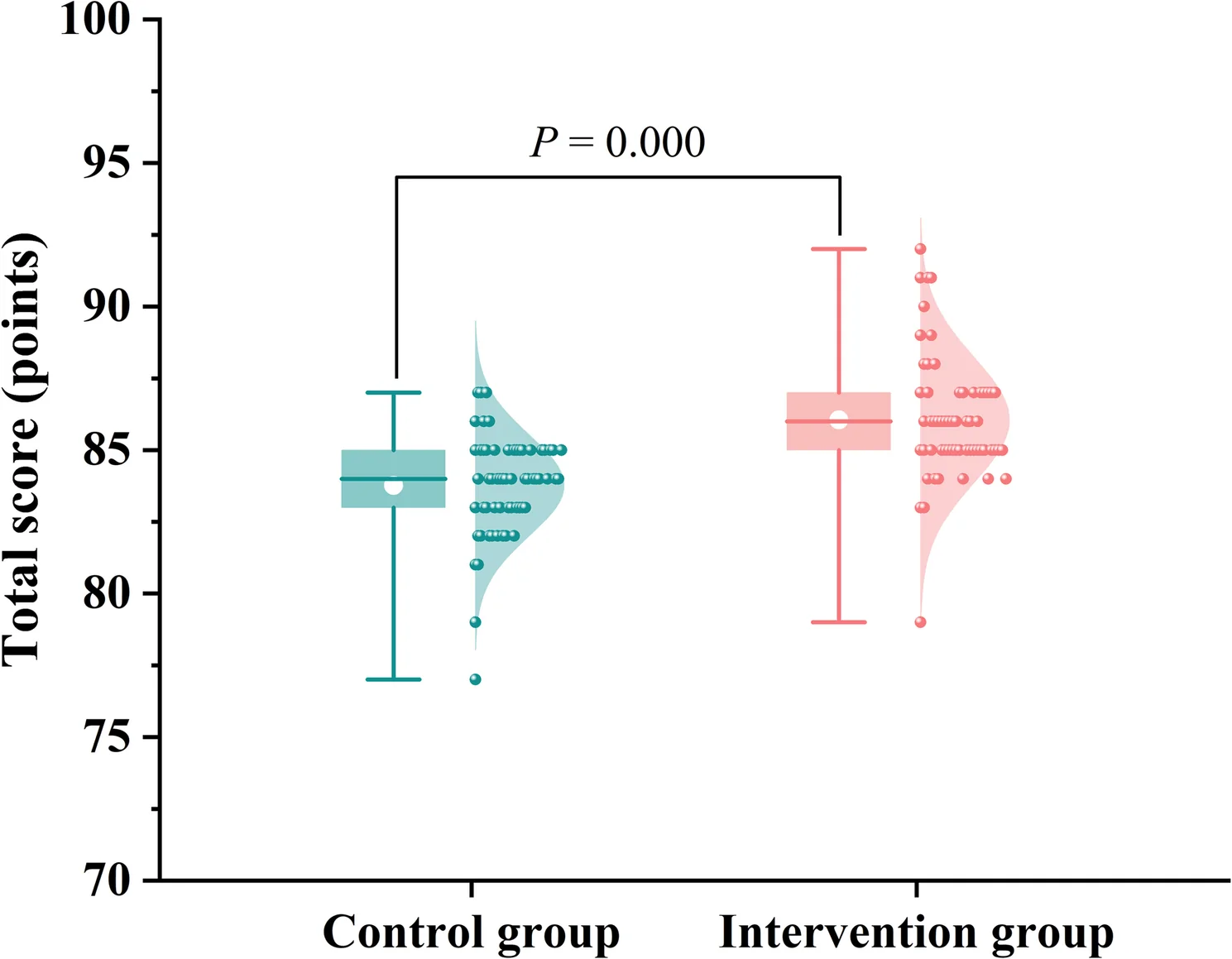
Comparative analysis of the experimental results between the control group and the intervention group

As shown in Fig. 2, the experimental scores of the intervention group (86.05 ± 2.166) were significantly higher than those of the control group (83.77 ± 1.764) (*t* = 6.351, *P* = 0.000). The between-group effect size Cohen’s *d* = 1.17, with 95% *CI* for mean difference [1.582, 2.768], indicating a large intervention effect.

### Analysis results of questionnaires

Students from the New Medical Science Class and Classes 1–2 of Medical Imaging participated in the survey and completed the questionnaires via the *Duifenyi* platform, achieving a 100% valid response rate. A comparative analysis of questionnaire results regarding epistemological beliefs, learning conceptions, and learning strategies between the control and intervention groups is presented in Table 3.

**Table 3:** Comparative analysis of questionnaire results between the control and intervention groups in terms of epistemological beliefs, learning conceptions, and learning strategies

Questionnaire	Project	Intervention group	Control group	t-value	*P*-value	Cohen’s d	95% CI for mean difference
SEQ	Stability of knowledge	17.59 ± 2.620	15.47 ± 2.324	4.892	0.000	0.844	[1.276,2.964]
	Structure of knowledge	25.52 ± 2.214	24.52 ± 2.867	2.259	0.026	0.388	[0.121,1.879]
	Omniscient authority	17.64 ± 2.679	16.10 ± 2.720	3.263	0.001	0.570	[0.607,2.743]
OLS	Preference for structure	23.43 ± 2.872	21.95 ± 2.854	2.96	0.004	0.518	[0.482,2.478]
	Preference for certainty	18.54 ± 2.958	16.35 ± 2.723	4.375	0.000	0.761	[1.223,3.157]
MLSQ	Surface learning strategy	15.62 ± 2.755	17.37 ± 2.836	-3.575	0.000	-0.623	[-2.734,-0.766]
	Deep learning strategy	23.64 ± 2.738	20.11 ± 2.954	7.087	0.000	1.230	[2.544,4.516]

#### SEQ

SEQ analysis demonstrated significantly higher overall scores in the intervention group on all three core dimensions (stability of knowledge, structure of knowledge, omniscient authority), with small-to-large between-group effect sizes (Table 3). All detailed dimensional statistics and item-level subscale comparisons are summarized in Table 4. The intervention group showed consistent advantages across nearly all sub-items covering curriculum design, knowledge system construction, and recognition of teaching guidance, with most differences statistically significant.

**Table 4:** Comparative analysis of the questionnaire results of epistemological beliefs between the control and intervention groups

Project	Intervention group	Control group	t-value	*P*-value	Cohen’s d	95% CI for mean difference
Stability of knowledge						
The knowledge listed in the experimental courseware is systematic.	3.65 ± 1.012	3.31 ± 0.781	2.201	0.030	0.377	[0.033,0.647]
If the experimental results are different from the experimental courseware, I usually analyze the experimental steps.	3.43 ± 1.022	3.32 ± 0.864	0.675	0.501	0.116	[-0.206,0.426]
I prefer to follow the steps in the courseware during the experiment.	3.67 ± 0.852	3.39 ± 0.930	1.796	0.075	0.309	[-0.027,0.587]
The knowledge system of preventive medicine experiment should be updated in time.	3.41 ± 1.062	2.92 ± 1.013	2.676	0.008	0.460	[0.129,0.851]
Under the background of new medicine, the conclusion of preventive medicine experiment needs to be discussed in depth.	3.43 ± 1.182	2.53 ± 1.051	4.597	0.000	0.791	[0.515,1.285]
Structure of knowledge						
The framework construction of preventive medicine experiment is suitable for students’ needs.	4.13 ± 0.567	3.55 ± 0.761	4.919	0.000	0.847	[0.343,0.817]
In the experimental course, I try to find the logical connection between experimental operations.	4.16 ± 0.609	4.02 ± 0.757	1.198	0.233	0.206	[-0.089,0.369]
It is believed that the problem can be better understood by comparing different experimental results.	4.28 ± 0.725	4.26 ± 0.767	0.133	0.895	0.023	[-0.277,0.317]
Understanding experimental phenomena needs to start from a multidisciplinary perspective.	4.38 ± 0.666	4.28 ± 0.581	0.889	0.376	0.153	[-0.123,0.323]
Experimental operation is helpful to internalize the knowledge of preventive medical experiments.	4.36 ± 0.542	4.34 ± 0.676	0.222	0.825	0.038	[-0.206,0.246]
Tend to organically link experimental knowledge with experimental operation.	4.22 ± 0.683	4.15 ± 0.698	0.598	0.551	0.103	[-0.154,0.294]
Omniscient authority						
The conclusions provided by teachers in experimental courses are relatively accurate.	3.74 ± 0.918	3.19 ± 0.920	3.392	0.000	0.583	[0.323,0.777]
Tend to rely on the experimental ideas in courseware.	3.71 ± 0.941	2.94 ± 0.903	4.795	0.000	0.824	[0.454,1.087]
Without the guidance of teachers, it is difficult for me to carry out the experimental operation.	3.33 ± 0.980	3.42 ± 0.950	-0.509	0.612	-0.088	[-0.431,0.251]
I believe more in the experimental data from authoritative sources, but I need to analyze it in combination with reality.	3.54 ± 1.008	3.21 ± 0.908	1.939	0.055	0.333	[-0.008,0.668]
I tend to regard the examples provided in the experimental courseware as the optimal solution.	3.37 ± 0.960	3.34 ± 0.922	0.175	0.861	0.030	[-0.303,0.363]

#### OLS

OLS analysis indicated that the intervention group achieved significantly higher scores on two core OLS dimensions, accompanied by small-to-moderate between-group effect sizes (Table 3). Full dimensional statistics and item-level subscale comparisons are provided in Table 5. Overall, the intervention group exhibited stronger structural learning preferences and greater demand for definite, authoritative experimental information across most sub-items, with most intergroup differences reaching statistical significance.

**Table 5:** Comparative analysis of the questionnaire results of learning conceptions between the control and intervention groups

Project	Intervention group	Control group	t-value	*P*-value	Cohen’s d	95% CI for mean difference
Preference for structure						
I like teachers to teach according to the syllabus.	3.97 ± 0.874	3.89 ± 0.832	0.561	0.576	0.096	[-0.182, 0.342]
The clear steps provided by the experimental courseware make it easier for me to complete the experimental task.	4.45 ± 0.557	3.69 ± 0.841	5.992	0.000	1.030	[0.500, 1.020]
I prefer structured experimental learning process to random exploration.	3.68 ± 0.947	3.73 ± 0.926	-0.272	0.789	-0.047	[-0.384, 0.284]
If the experimental courseware does not clearly point out the key content, I will feel confused.	3.81 ± 0.912	3.74 ± 0.867	0.447	0.656	0.077	[-0.230, 0.370]
When the experimental objectives are listed in the experimental courseware, it is easier for me to concentrate on my study.	4.36 ± 0.593	3.98 ± 0.878	2.917	0.004	0.502	[0.147, 0.693]
I hope that all experimental problems have a unified and correct solution.	3.16 ± 1.038	2.92 ± 1.121	1.273	0.205	0.219	[-0.130, 0.590]
Preference for certainty						
I don’t like to ask questions that are answered from different angles.	3.28 ± 1.042	2.90 ± 1.082	2.005	0.047	0.345	[0.005, 0.555]
I prefer experiments with fixed operation steps.	3.14 ± 1.047	2.97 ± 1.008	0.984	0.327	0.169	[-0.172, 0.512]
If there are contradictory opinions in the experiment, I tend to ask the teacher to provide a clear explanation.	4.14 ± 0.733	3.60 ± 0.914	3.760	0.000	0.647	[0.257, 0.823]
During the experiment, I hope to know whether my experiment is correct as soon as possible.	3.99 ± 0.962	3.40 ± 1,047	3.301	0.001	0.568	[0.231, 0.949]
I feel uneasy about the ambiguous information during the experiment.	3.99 ± 0.899	3.48 ± 1.004	2.999	0.003	0.516	[0.161, 0.859]

#### MLSQ

MLSQ results revealed contrasting group differences across the two learning strategy dimensions (Table 3). The control group earned significantly higher scores on surface learning strategies, while the intervention group demonstrated markedly superior deep learning strategy performance, with moderate between-group effect sizes for both outcomes (both exact *P* = 0.000). Full item-level comparative statistics are presented in Table 6.

**Table 6:** Comparative analysis of the questionnaire results of the learning strategies between the control and intervention groups

Project	Intervention group	Control group	t-value	*P*-value	Cohen’s d	95% CI for mean difference
Surface learning strategy						
Before exams, I always try my best to memorize knowledge.	3.91 ± 1.095	4.50 ± 0.621	-3.822	0.000	-0.658	[-0.904,-0.276]
When I encounter a problem, I don’t understand, I am used to skipping it instead of delving into it.	2.59 ± 0.896	2.76 ± 1.066	-0.955	0.341	-0.164	[-0.527,-0.187]
When completing the experimental report, I mainly rely on the sample templates in the experimental courseware.	3.32 ± 0.915	3.71 ± 0.876	-2.490	0.014	-0.429	[-0.708,-0.092]
When standard answers are provided in the experimental courseware, I seldom explore other possible solutions.	3.12 ± 0.963	3.24 ± 1.097	-0.700	0.485	-0.121	[-0.464, 0.224]
I prefer to remember the new knowledge in the experiment first rather than try to understand it.	2.68 ± 1.231	3.16 ± 1.190	-2.265	0.025	-0.390	[-0.902,-0.068]
Deep learning strategy						
When studying, I will contact my own problems and think and question the theory.	3.77 ± 0.770	3.42 ± 0.841	2.479	0.014	0.427	[0.075, 0.625]
During the experiment, I will try many times to get the exact result.	4.09 ± 0.935	3.40 ± 0.966	4.113	0.000	0.708	[0.361, 1.019]
I will take the initiative to ask questions that are not involved in the experiment and try to find answers.	3.54 ± 0.964	3.03 ± 0.886	3.103	0.002	0.534	[0.175, 0.845]
I tend to combine the experimental content with my existing knowledge for thinking.	4.01 ± 0.776	3.44 ± 0.802	4.188	0.000	0.721	[0.285, 0.855]
When learning experimental knowledge, I pay more attention to the logical connection between them than to a single knowledge point.	4.28 ± 0.591	3.60 ± 0.931	4.915	0.000	0.846	[0.399, 0.961]
Facing the open problem in the experiment, I will think about several possible solutions.	3.96 ± 0.830	3.23 ± 0.965	4.620	0.000	0.795	[0.405, 1.055]

#### Structural equation modeling analysis of the relationships among epistemological beliefs, learning conceptions, and learning strategies

The zero-order correlation matrix in Table S2 demonstrated significant positive correlations between surface learning strategies and both epistemological beliefs (knowledge structure, stability, and omniscient authority) and learning conceptions (structural/certainty preferences). While knowledge stability and structural preference were significantly positively associated with deep learning strategies, omniscient authority, and certainty preference were significantly negatively correlated with deep learning strategies, with the negative relationship between knowledge structure and deep learning strategies remaining statistically non-significant.

**Fig. 3:**
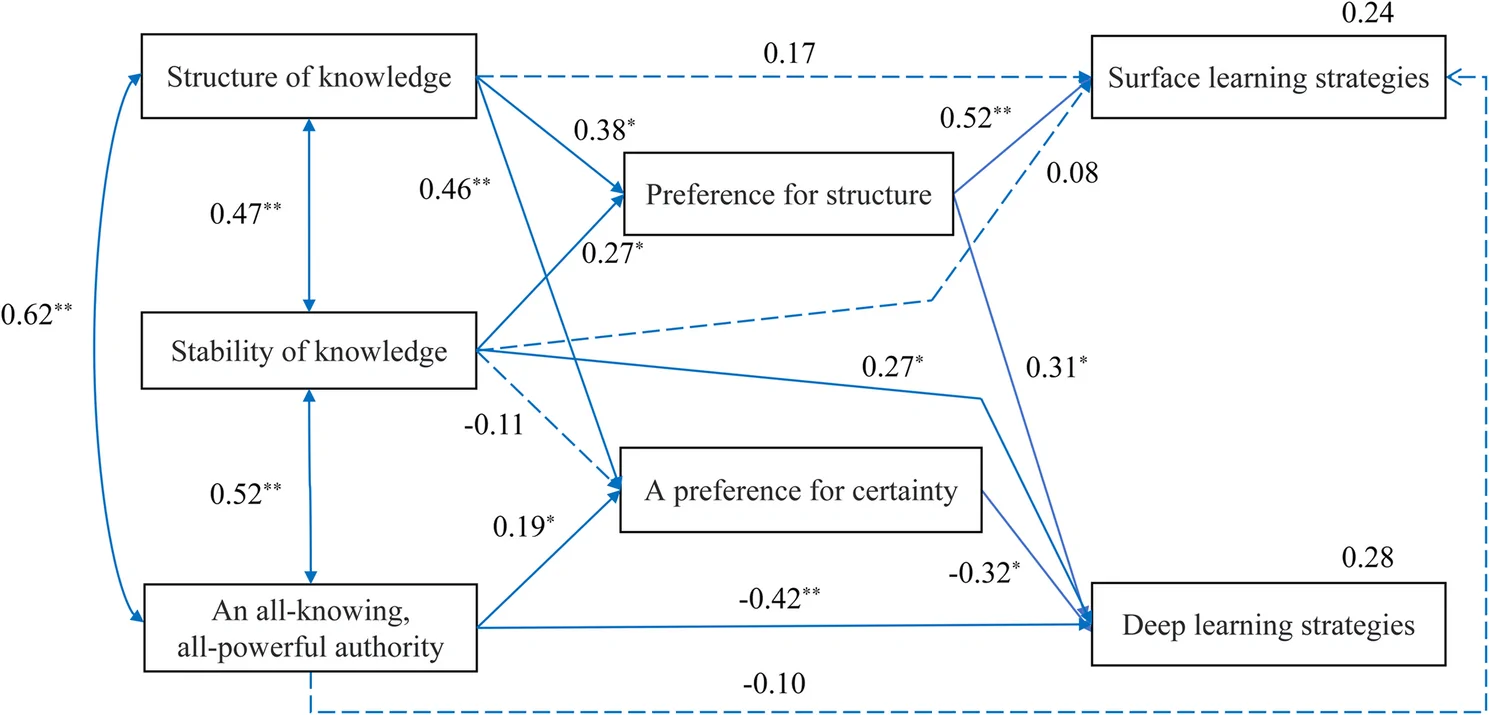
Path map of structural equation modeling analysis of the relationships among epistemological beliefs, learning conceptions, and learning strategies. (*: *P* < 0.05; **: *P* < 0.01)

The adjusted structural equation model demonstrated acceptable model fit (*χ*²*/df* = 2.12, RMSEA = 0.071, NNFI = 0.556, CFI = 0.888), as illustrated in Fig. 3. Epistemological beliefs and learning conceptions collectively accounted for 24% of the variance in surface learning strategies. Among these, structural preference (*β* = 0.52) significantly predicted surface strategies, while epistemological beliefs did not exert direct effects. Knowledge structure and knowledge stability indirectly influenced surface learning through the mediating role of structural preference, whereas certainty preference and omniscient authority did not demonstrate significant mediation pathways within this model configuration. This finding aligns with the core feature of learning plan guidance: the structured pre-class learning scaffolds shape students’ preference for structured learning, which may manifest as procedural replication (typical surface learning). Additionally, epistemological beliefs and learning conceptions explained 28% of the variance in deep learning strategies. Knowledge stability (*β* = 0.27) and structural preference (*β* = 0.31) emerged as significant positive predictors, whereas omniscient authority (*β* = − 0.42) and certainty preference (*β* = − 0.32) were significant negative predictors. These results reveal the internal mechanism of the BOPPPS-driven learning plan model: pre-class learning plans build a stable, structured knowledge base to support deep learning, while the participatory learning and real-time feedback loops of BOPPPS weaken students’ belief in omniscient authority and preference for deterministic knowledge, jointly driving the adoption of deep learning strategies. Knowledge stability contributed both direct effects and indirect positive mediation through structural preference, while omniscient authority exerted dual influences via direct negative effects and indirect mediation through the reduction of certainty preference.

Table 7 shows the significant mediating effects of structural preference between knowledge structure/stability and learning strategies and of certainty preference between knowledge structure and deep learning strategies. Specifically, knowledge structure exerted a positive influence on both surface learning strategies (indirect effect = 0.20) and deep learning strategies (indirect effect = 0.12) through the mediating effect of structural preference (*β* = 0.38). The dual-direction effect of structural preference further verifies the complementary value of the integrated pedagogy: the learning plan provides necessary structural support for learning, while BOPPPS in-class sessions guide students to move beyond surface-level procedural compliance and apply structural knowledge to deeper cognitive processing. Concurrently, it negatively impacted deep learning strategies via mediation through certainty preference (*β* = 0.46, indirect effect = − 0.15). Knowledge stability demonstrated indirect positive effects on surface learning strategies (indirect effect = 0.14) and deep learning strategies (indirect effect = 0.08) by enhancing structural preference (*β* = 0.27).

**Table 7:** Significance test of the mediation effects using MCMAM analysis

Path	Standardized indirect effect estimate	95% Confidence interval
Structure of knowledge → Preference for structure → Surface learning strategy	0.38 × 0.52 = 0.20	[0.13, 0.44]
Structure of knowledge → Preference for structure → Deep learning strategy	0.38 × 0.31 = 0.12	[0.09, 0.29]
Structure of knowledge → Preference for certainty → Deep learning strategy	0.46 × (-0.32) = -0.15	[-0.37, -0.07]
Stability of knowledge → Preference for structure → Surface learning strategy	0.27 × 0.52 = 0.14	[0.11, 0.39]
Stability of knowledge → Preference for structure → Deep learning strategy	0.27 × 0.31 = 0.08	[0.02, 0.19]
Omniscient authority → Preference for certainty → Deep learning strategy	0.19 × (-0.32) = -0.06	[-0.18, 0.01]

#### TSES

TSES questionnaire data demonstrated obvious performance gaps across three subscales of student management, teaching strategy implementation and classroom management (Table 8). Teachers reported the highest self-efficacy in evaluating learning mastery, cultivating students’ innovation and entrepreneurship, and handling unexpected learning difficulties. By contrast, relatively low confidence was observed in differentiated teaching tasks, including supporting underachieving students, adjusting teaching pace for individual differences, applying diversified teaching strategies and regulating classroom atmosphere. All detailed item scores within each dimension are fully presented in Table 8.

**Table 8:** Analysis of the questionnaire results of teaching efficacy for the intervention group of students

Project	Scores
Student Management	
Encourage students who do not enjoy attending classes to do so as much as possible	3.06 ± 0.854
Assist students in critical thinking	3.62 ± 0.806
Motivate students who are not interested in learning	3.19 ± 0.750
Make students believe that they can do better in their studies	3.19 ± 0.834
Cultivate students’ innovation and entrepreneurship	4.25 ± 0.683
Improve the comprehension ability of failing students	2.31 ± 1.195
Implementation of teaching strategies	
Answer the difficult questions raised by students	3.81 ± 0.655
Measuring students’ understanding of the taught content	4.44 ± 0.629
Design good questions for students	3.50 ± 0.894
Adjust the course schedule to suit each student’s level	2.31 ± 0.793
Use formative assessment	3.37 ± 0.719
Provide another explanation when students feel confused	3.25 ± 0.775
Implement alternative strategies in the classroom	2.38 ± 1.310
Provide advanced challenges for students with strong abilities	3.94 ± 0.680
Classroom management	
Control the classroom learning atmosphere well	2.31 ± 0.793
Clarify expectations for students’ behavior	3.37 ± 0.719
Establish rules to ensure smooth teaching progress	3.25 ± 0.775
Encourage students to follow the rules of the project	2.38 ± 1.310
Soothing emotionally unstable students	3.94 ± 0.680
Establish a good project management connection with each group of students	3.19 ± 0.750
Dealing with unexpected problems in students’ learning	4.25 ± 0.856

## Discussion

This study examines the implementation of BOPPPS-driven learning plan guidance in preventive medicine laboratory education, utilizing comparative analyses of academic performance and questionnaire data between intervention and control groups. The results demonstrated that this instructional model significantly improved academic performance, learning conceptions, learning strategies, and teachers’ self-efficacy within the context of preventive medicine laboratory instruction.

### Optimization of experimental teaching content—dual enhancement of practical skills and academic performance

This investigation revealed the consistent superiority of the intervention group across all the metrics through a comparative analysis of experimental reports and questionnaire data, demonstrating the pedagogical efficacy of the BOPPPS-driven learning plan guidance model. Compared with conventional models, quantitative improvements in student engagement, critical thinking, and instructional outcomes substantiate its advantages. The structured framework of this model fosters self-regulated learning by promoting systematic pre-class preparation and post-class consolidation. It enhances discipline-specific motivation and goal-oriented learning behaviors through the explicit articulation of learning objectives, thereby contributing to improved academic performance [10]. Comparative analysis underscores the pedagogical limitations of traditional instructional approaches, as evidenced by memory retention rates below 30% among students. In contrast, BOPPPS-structured models that incorporate cooperative, active, and participatory learning paradigms demonstrate superior efficacy. The systematic implementation of learning plans and cooperative activities facilitates deeper content assimilation, with memory retention rates exceeding 50%. Participatory learning serves as the cornerstone of this model, significantly enhancing students’ practical competencies. Moreover, the model enables educators to guide problem-based knowledge co-construction through structured discourse, thereby improving both instructional effectiveness and student academic outcomes [9]. Successful implementation of this teaching model also requires educators to possess expertise in curricular design and instructional delivery, along with the ability to continuously monitor learner progression, provide real-time feedback, and adapt teaching strategies for optimal pedagogical outcomes.

### Innovative teaching model—reshaping students’ learning conceptions

Learning conceptions represent students’ foundational cognitive schemata that govern knowledge acquisition processes, evolving through the systematic examination of epistemic constructs, multidimensional valuation of learning orientations, and discursive pluralism in knowledge construction. OLS analyses indicated that students exposed to BOPPPS-driven learning plan guidance displayed a marked preference for structured and predictable classroom content, as well as topic-specific knowledge characterized by definitive answers and low ambiguity during the learning process. Epistemological beliefs exert a significant influence on students’ selection of learning strategies, wherein structured knowledge architectures facilitate the development of organizational strategies, and systematic engagement with content fosters critical thinking competencies [30]. Notably, beliefs concerning the certainty of knowledge function as key mediators within self-regulated learning mechanisms, consistent with the findings reported by Bråten and Strømsø [31]. With respect to certainty preference, the intervention group of students exhibited a stronger inclination toward explicit experimental guidance and explanations, which was correlated with the teaching model’s emphasis on knowledge certainty and standardization. This disposition also reveals decreased autonomous problem-solving initiatives in ambiguous contexts, necessitating targeted instructional interventions that balance structured guidance with the development of exploratory competencies are essential. This teaching model effectively reshapes students’ learning conceptions by prioritizing engagement with logically organized, outcome-defined content. Such an approach reinforces domain-specific competency acquisition and promotes strategic learning optimization, ultimately cultivating autonomous learners endowed with heightened analytical rigor and interdisciplinary proficiency [32].

### Transformation of the evaluation system—new guidelines for learning strategies

This pedagogical synthesis illustrates the efficacy of the innovative model in enhancing knowledge comprehension and learning strategies, while simultaneously activating critical analysis and self-regulated learning capacities. Rather than merely emphasizing the final results of experimental reports, the evaluation system employed in this study utilizes a three-dimensional weighted framework—experimental completion (60%), result analysis (20%), and report standardization (20%)—which inherently aligns with the post-assessment stage of the BOPPPS model. This approach is intended to provide process-based assessment of students across various dimensions, such as procedural compliance, analytical reasoning, and expository clarity. Comparative analysis of experimental report metrics confirmed that students in the intervention group exhibited improved critical thinking and practical competencies. Additionally, the model enhanced adaptive problem-solving during experimental contingencies, thereby demonstrating its effectiveness in cultivating holistically skilled professionals through structured didactic innovation. Findings from the SEQ further validated the model’s effectiveness in fostering student recognition of the imperatives of knowledge system modernization and encouraging prioritized analytical engagement with experimental outcomes in laboratory settings. These results align with existing research indicating that sophisticated epistemological frameworks are positively associated with improved educational outcomes in medical training [33]. Given that medical education frequently involves complex, ill-structured problems that defy algorithmic resolution, students must integrate newly acquired knowledge with existing cognitive frameworks to apply contextually appropriate solutions in dynamic clinical scenarios [34].

Furthermore, analyses from the MLSQ confirm the pedagogical approach’s effectiveness in promoting the proactive adoption of deep learning strategies through problem-based engagement that stimulates critical analysis and theoretical inquiry. This improvement can be fully explained by the staged instructional design of the six BOPPPS components. At the Bridge-in stage, scenario-based preview tasks embedded in learning plans activate students’ prior knowledge and raise experimental questions that trigger cognitive conflict; unlike conventional preview that only requires reading operational procedures, this design eliminates rote memorization and establishes active inquiry as the starting point of deep learning. Explicit multi-dimensional competency objectives guide students to focus on internal logical connections within preventive medicine experiments, rather than memorizing isolated operational steps, while pre-class formative pre-assessments promptly identify knowledge gaps and encourage students to integrate new and prior knowledge before formal laboratory operations. Participatory learning serves as the core driver of the transition from surface-level learning to deep cognition: group collaborative case discussions, such as distinguishing infectious and toxic food poisoning, unresolved questions recorded on discussion sheets for teacher review, and random group presentations prompt students to propose hypotheses, conduct multi-angle comparative analyses and correct subjective biases through communication, which are core behavioral markers of deep learning. The post-assessment evaluation system combines lab reports with online quizzes on the *Duifenyi* platform to strengthen appropriate transfer processing, reduces the scoring weight of standard answers and increases the proportion of result discussion and logical deduction to consolidate critical thinking habits, and in the final Summary stage, instructors adopt mind maps to organize systematic knowledge frameworks for long-term integrated memory instead of fragmented short-term knowledge retention.

Accordingly, the BOPPPS-driven learning plan guidance pedagogical model employs learner-centric didactics, strategically adapting instructional sequences to learner profiles through mechanisms such as contextual knowledge integration via the bridge-in phase, enabling cross-contextual concept application and cognitive consolidation [35]. The systematic integration of learning plan guidance with structured curricular content organization establishes a cohesive knowledge architecture that enhances students’ cognitive assimilation and retention, thereby improving subject matter proficiency through increased conceptual clarity and information consolidation. The BOPPPS framework’s focus on participatory engagement and structured feedback mechanisms enhance learner satisfaction while cultivating critical thinking competencies [15]. Additionally, the model’s integration of predefined objectives with pre-assessments supports multi-modal self-regulated learning by facilitating exploratory research, autonomous analysis, and collaborative discourse—effectively transitioning learning strategies from surface-level memorization to deep cognitive processing [36]. In summary, the evaluation system of this study not only serves as a tool for measuring learning outcomes, but also functions as a pedagogical intervention. Through its synergistic interaction with the BOPPPS structural process, it simultaneously facilitates improvements in experimental performance and promotes the balanced development of students’ critical thinking and self-constructed learning abilities.

### Influence of epistemological beliefs on learning strategies—mediating role of learning conceptions

#### Predictive effects of epistemological beliefs and learning conceptions on learning strategies

Empirical studies demonstrate that omniscient authority inversely predicts deep learning strategies, whereas knowledge stability has positive predictive effects. Learners endorsing knowledge authoritarianism show a reduced propensity for deep learning strategies, a phenomenon observed by Klopp et al. with attenuated impacts among participants possessing sufficiently developed epistemological beliefs [37]. Conversely, Kienhues et al. reported enhanced epistemological sophistication when students confronted unstable knowledge systems [38], paralleling Richards et al.’s findings that ICU practitioners developed superior clinical reasoning capacities through exposure to complex knowledge environments [39].

Empirical analyses further indicate that structural preference serves as a positive predictor of both deep and surface learning strategies, whereas certainty preference exhibits a negative correlation with deep learning strategies. Harlow et al. reported that learners with a strong structural orientation tend to employ a diverse array of learning strategies [40], a finding supported by Feinkohl et al., who observed enhanced critical analysis during the processing of conflicting information [41]. In contrast, students with a high certainty preference demonstrate a marked aversion to deep learning approaches, favoring the assimilation of unambiguous content over the integrative knowledge synthesis that necessitates critical interrogation.

#### Mediating role of learning conceptions in the impact of epistemological beliefs on learning strategies

Learning conceptions function as a mediating variable between epistemological beliefs and learning strategies, with knowledge structure and stability influencing learning strategy selection through cognitive orientations toward certainty and structural clarity. The analysis reveals that knowledge structure may inhibit the adoption of deep learning strategies by reinforcing certainty-seeking tendencies. Learners who perceive knowledge as static and autonomous exhibit a heightened orientation toward certainty—an outlook fundamentally misaligned with the critical interrogation and integrative processing required for deep learning. This finding is supported by Chen et al., who identified behavioral inertia in certainty-oriented students during problem-solving tasks, marked by reduced strategic diversity and decreased academic performance [42].

The analyses further demonstrate that knowledge structure and stability enhance structural preference, thereby facilitating the adoption of both deep and surface learning strategies. Learners who perceive knowledge as compartmentalized yet reliable tend to exhibit a dependency on structured instruction and often face difficulties integrating knowledge when such strategies are not present. This innovative pedagogical model, through systematized knowledge integration, facilitates students’ in-depth comprehension of the essential characteristics of knowledge while aligning with their structured cognitive demands for a systematic knowledge framework. Heimbuch et al. identified the need for cognitive closure as significantly associated with individual differences in learning and knowledge construction [43], while Gärtner et al. underscored its critical relevance for medical students confronting clinical uncertainty [44]. Accordingly, the BOPPPS-based learning plan guidance model in medical education has the potential to modulate students’ certainty and structural preferences, thereby optimizing the implementation of deep learning strategies while maintaining a strategic balance between surface and deep learning approaches [45].

### Empowerment of innovative teaching model—advancement of teacher efficacy

Analyses of the TSES questionnaire revealed broad educator consensus that the BOPPPS-integrated learning plan guidance model enhances student management, instructional strategy implementation, and classroom governance. It was also recognized for effectively fostering students’ innovative capacities, facilitating content comprehension assessment, and addressing emergent learning issues. This pedagogical approach received high approval ratings despite its demand for elevated instructional competencies, as it redefines the educator’s role from a knowledge transmitter to a multifaceted facilitator. Successful implementation of the model necessitates instructors’ proficiency in operationalizing its structured-interactive framework [46]. However, the questionnaire results also indicated lower approval ratings in areas such as improving comprehension among underperforming students, adapting instructional pacing, and regulating classroom climate. These shortcomings were attributed to two primary factors: students’ mechanical execution of learning plans without critical engagement or collaborative skill development, and instructors’ emphasis on overall class progression at the expense of individualized monitoring. Pedagogical recommendations include the real-time adjustment of instructional pacing based on vigilant observation of student engagement, along with the incorporation of enhanced interactive scaffolding to address challenges in autonomous learning. Additionally, fostering structured peer interaction is crucial for counteracting task-focused rigidity and promoting a dynamic, collaborative learning environment that supports optimal educational outcomes and classroom vitality.

### Limitations and future directions

The empirical validation conducted in this investigation affirms the multidimensional pedagogical efficacy of the BOPPPS-driven learning plan guidance model in preventive medicine laboratory education, while also identifying persistent implementation challenges and methodological limitations warranting further consideration. Primarily, the study’s methodological constraints include limited generalizability due to its single-institution design, restricted sample size, and short intervention duration—all of which may have attenuated the observable impact of the model’s demonstrated advantages. Additionally, though participants were recruited from two distinct majors, the two cohorts completed identical preclinical prerequisite courses and demonstrated well-balanced baseline characteristics (Table 2, all *P* > 0.05). Nevertheless, students from the two majors perceive distinct functional roles of preventive medicine courses. Accordingly, future multi-center trials that recruit larger samples within a single major are necessary to strengthen the external validity of the present findings across various medical education settings. Second, the experimental processes exhibited learner passivity characterized by rote completion of learning modules, superficial cognitive engagement, and inadequate group collaboration. Inter-student variability in learning motivation and competence constitutes a critical source of heterogeneity [8]. Such insufficient learning capabilities might undermine the pedagogical advantages of this instructional model while compromising experimental validity through impairing knowledge co-construction dynamics. Third, instructors’ emphasis on adhering to curricular schedules contrasts with the learner-centered, closed-loop pedagogical architecture employed by this model, which prioritizes participatory engagement and iterative feedback mechanisms. Insufficient attention to student input by educators adversely affects the learning efficacy of certain students by limiting the effective integration of feedback into the instructional process.

Future educational research initiatives will implement progressive refinements to address these challenges. First, educators should enhance their responsiveness to student feedback to support ongoing pedagogical optimization and improve learning quality. Second, systematic experimentation with the BOPPPS-driven learning plan guidance model will generate more granular and actionable insights, while fostering students’ autonomous critical thinking and collaborative competencies to fully realize the model’s instructional benefits. Moreover, innovative integration with complementary pedagogical frameworks, such as hybrid PBL approaches [47], will form a critical trajectory for future research. Although not yet ubiquitous, this student-centered closed-loop pedagogical framework has substantial potential for advancing preventive medicine education and multidisciplinary instruction, aligning with modern competency-based talent cultivation paradigms through sustained academic exploration and practical implementation. Finally, the cluster-randomized design may produce intra-cluster correlation and reduce statistical power. Future research could enlarge the sample size and adjust statistical models to control the ICC effect for more robust results.

## Conclusion

This investigation of the implementation of the BOPPPS-driven learning plan guidance strategy in preventive medicine laboratory education demonstrated enhanced multidimensional outcomes, including performance metrics, learning motivation, and self-regulated learning capacity. Furthermore, educator evaluations confirmed significant improvements in instructional strategy execution, classroom governance, and learner management, collectively enhancing overall pedagogical efficacy. The dual emphasis of this framework on consolidating foundational knowledge and developing applied competencies in public health emergency response carries critical implications for contemporary medical education reform. These multidimensional outcomes highlight the framework’s potential to cultivate cognitively agile professionals capable of addressing evolving healthcare challenges, thereby warranting expanded longitudinal studies to assess its scalability across diverse medical disciplines.

## Supplementary Information

Supplementary Material 1.

## Data Availability

Data is provided within the manuscript or supplementary information files.
