# Supplementary material for: BOPPPS-driven learning plan guidance in preventive medicine laboratory education: Implementation strategy and multidimensional outcomes
Source: BMC Med Educ. 2026 Jul 28;26:1717. doi: 10.1186/s12909-026-10038-8 (PMC13700289; doi:10.1186/s12909-026-10038-8)
Supplement: Supplementary file 1 [file 12909_2026_10038_MOESM1_ESM.docx]

**Supplementary Material**

**Operational workflow of BOPPPS-driven learning plan guidance pedagogy in preventive medicine laboratory education:**

**Bridge-in (B) (15 min)**

Before class, instructors developed comprehensive learning plans comprising six components: conceptual mind maps outlining experimental knowledge, interdisciplinary relevance diagrams, learning content, instructional resources, pedagogical strategies, and clearly defined learning objectives. Standardized discussion record sheets were also prepared to help students document pre-class discussions and unresolved questions. All learning materials were electronically distributed to students one week before the experimental session. Students in the intervention group were assigned to heterogeneous learning groups (4–5 students per group) via stratified grouping based on pretest scores, academic performance, gender, and learning attitudes. Baseline comparisons confirmed no statistically significant differences across learning groups (all *P* > 0.05), ensuring balanced group homogeneity before intervention.

This pre-class and in-class bridge-in stage lasted 15 minutes and contained structured individual and collaborative preparation. For individual self-study, each student independently studied the learning plan, supplemented mind map notes, and sorted out confusing knowledge points. For synchronous online group discussion, group members exchanged preliminary understandings, debated difficult content, and jointly completed discussion record sheets to document group consensus and unsolved questions. Three days before class, each group representative uniformly collected individual learning plans and group discussion sheets and submitted all materials to the instructor.

In this stage, instructors systematically reviewed pre-submitted materials, summarized common knowledge gaps and typical misconceptions reflected in pretest performance and discussion records, and adjusted in-class teaching emphasis accordingly. No real-time one-on-one tutoring was provided during pre-class self-study; collective confusion was reserved for targeted clarification during formal instruction.

**Objective (O) (10 min)**

Instructors explicitly presented hierarchical learning objectives aligned with the BOPPPS framework and modern medical education requirements for competent, skilled, and ethical public health practitioners. Based on the food poisoning case scenario, three-dimensional learning objectives were clearly stated. Knowledge objectives: Consolidate foundational epidemiological and microbiological knowledge to accurately identify food poisoning events and formulate standard public health emergency response protocols. Competency objectives: Master field sampling, on-site investigation, and emergency disposal procedures for food poisoning incidents and improve practical public health response capabilities. Literacy objectives: Integrate medical humanistic care and professional ethics into case analysis to cultivate students’ sense of professional responsibility and integrity.

This stage adopted teacher-led instruction with no group tasks. During objective interpretation, students individually marked personal learning gaps on their own learning plans according to the stated objectives. Instructors mainly connected pre-class preparation content with in-class learning goals, clarified the logical correspondence between subsequent teaching activities and learning outcomes, and helped students establish clear learning expectations.

**Pre-assessment (P) (15 min)**

The pre-assessment segment lasted 15 minutes and combined quantitative pretest score analysis and qualitative in-class questioning to evaluate students’ baseline knowledge level. All students completed a unified online pretest before class, focusing on experimental principles, operational procedures, and food poisoning-related microbiological and epidemiological knowledge. Pretest scores were used as objective baseline data to reflect students’ prior academic proficiency. In class, instructors conducted random spot checks and targeted whole-class questioning to verify pre-class preparation quality and cross-validate pretest results.

All pretest completion and in-class questioning were completed independently by individual students, and students voluntarily shared difficult knowledge points summarized during pre-class preparation. No collaborative group activities were arranged in this stage. Instructors analyzed the overall distribution of pretest scores, identified widespread knowledge vulnerabilities and potential misconceptions, and dynamically adjusted the teaching depth and difficulty of the subsequent participatory learning session based on real-time student performance.

**Participatory learning (P) (60 min, core session)**

Participatory learning was the core instructional segment, occupying 60 minutes of the 120-minute class. All activities were clearly divided into individual independent practice and collaborative group tasks with standardized time allocation and organizational rules. Group discussion and presentation preparation (15 min, collaborative): Fixed groups of 4–5 students revised and optimized case analysis frameworks and summarized key knowledge concerning food poisoning classification, emergency disposal, and on-site investigation based on pre-class discussion records. Each group appointed a rotating moderator to ensure equal member participation and avoid dominant individual discourse. Discussion processes were audio-recorded and photographically documented.

Random group presentation and peer Q&A (20 min, collaborative output): Instructors randomly selected groups to present their case analysis and experimental design. After each 3–5 min group report, a 3-minute open class Q&A was conducted, allowing peers to raise questions, provide supplements, and conduct peer evaluation. Targeted teacher clarification and misconception correction (15 min): Instructors centrally explained common problems summarized from pretest results, pre-class discussion sheets, and in-class peer communication. Typical misconceptions (e.g., confusion between infection-type and toxin-type food poisoning) were resolved through systematic interpretation of bacterial characteristics, exotoxins, and endotoxin mechanisms.

On-site experimental operation training (10 min, mixed individual and group tasks): Basic operational steps were practiced individually; field simulation of food, vomitus, and feces sampling and environmental investigation were completed through group collaboration. During the whole session, instructors circulated among groups for real-time observation, provided heuristic prompts to facilitate stagnant discussions, delivered instant verbal feedback during operational practice, and recorded persistent student misconceptions that could not be resolved through peer interaction. Targeted heuristic guidance was adopted instead of directly providing standard answers.

**Post-assessment (P) (15 min)**

Post-assessment lasted 15 minutes and was designed to evaluate in-class learning outcomes immediately after participatory activities. All tasks were completed independently without group cooperation. Each student independently completed a preliminary experimental report framework and finished online knowledge quizzes released via the *Duifene* platform. Composite post-intervention scores were generated based on weighted experimental report performance and online quiz results to quantitatively reflect learning improvement.

In this stage, instructors monitored online quiz completion progress in real time, provided on-site verbal reminders for obvious logical or operational errors in students’ draft reports, and sorted out typical problems for subsequent centralized feedback.

**Summary (S) (5 min)**

The final in-class summary lasted 5 minutes. Instructors synthesized multidimensional learning evidence, including pretest baseline performance, learning plan completion quality, group discussion records, in-class presentation performance, and post-assessment scores. Combined with the knowledge mind map in the learning plan, instructors systematically sorted out core knowledge frameworks, key operational procedures, and common student errors, helping students consolidate and internalize learned content. After class, students independently revised and finalized complete experimental reports and submitted formal versions within three working days.

Instructors graded all reports according to standardized evaluation criteria (**Table S1**) and provided individualized written feedback for each student. Universal common errors were summarized and announced in the next class. Students could submit unresolved questions via the online platform after class, and instructors provided unified centralized responses to eliminate residual knowledge misunderstandings, forming a closed-loop teaching feedback and misconception correction mechanism.

**Table S1.** Evaluation criteria for experimental reports of students in the control group and experimental group.

| **Score range** | **Completion of experiments**  **(60%)** | **Analysis of experimental results**  **(20%)** | **Standardization of reports**  **(20%)** |
| --- | --- | --- | --- |
| **90-100** | Full completion with detailed steps and accurate results. | Strong logic, clearly demonstrating connections. | Neat, standardized, and adheres to academic integrity. |
| **80-89** | Most completion with relatively complete steps. | Logical, addressing main connections. | Generally neat and compliant with basic standards. |
| **70-79** | Partial completion; Steps require refinement. | Moderate logic, fails to clearly demonstrate connections. | Moderate neatness and standardization. |
| **60-69** | Low completion; Significant improvement needed. | Lacks logic and fails to effectively demonstrate connections. | Poor neatness and standardization. |

**Table S2.** Zero-order correlation matrix of cognitive beliefs, learning views and learning strategies.

| **Dimension** | **1** | **2** | **3** | **4** | **5** | **6** | **7** |
| --- | --- | --- | --- | --- | --- | --- | --- |
| **1 Structure of knowledge** | 1 |  |  |  |  |  |  |
| **2 Stability of knowledge** | 0.318^*^ | 1 |  |  |  |  |  |
| **3 all-knowing and all-powerful authority** | 0.407^**^ | 0.761^**^ | 1 |  |  |  |  |
| **4 Preference for structure** | 0.352^*^ | 0.422^**^ | 0.357^**^ | 1 |  |  |  |
| **5 A preference for certainty** | 0.488^**^ | 0.224^*^ | 0.469^**^ | 0.526^**^ | 1 |  |  |
| **6 Surface learning strategy** | 0.259^*^ | 0.312^*^ | 0.187^*^ | 0.541^**^ | 0.489^**^ | 1 |  |
| **7 Deep learning strategy** | -0.07 | 0.444^**^ | -0.425^**^ | 0.525^**^ | -0.324^*^ | 0.247^*^ | 1 |

*: *P* < 0.05; **: *P* < 0.01.
